# Pestalotiones A–D: Four New Secondary Metabolites from the Plant Endophytic Fungus *Pestalotiopsis Theae*

**DOI:** 10.3390/molecules25030470

**Published:** 2020-01-22

**Authors:** Longfang Guo, Jie Lin, Shubin Niu, Shuchun Liu, Ling Liu

**Affiliations:** 1 State Key Laboratory of Mycology, Institute of Microbiology, Chinese Academy of Sciences, Beijing 100101, China; glf533@126.com (L.G.); liusc@im.ac.cn (S.L.); 2 University of Chinese Academy of Sciences, Beijing 100039, China; 3 Jiangsu Key Laboratory for Biofunctional Molecules, College of Life Science and Chemistry, Jiangsu Second Normal University, Nanjing 210003, China; linjie@jssnu.edu.cn; 4 School of Biological Medicine, Beijing City University, Beijing 100083, China; niushubin0704@163.com

**Keywords:** structure elucidation, secondary metabolites, *Pestalotiopsis theae*, cytotoxicity, antioxidant

## Abstract

Two new xanthone derivatives, pestalotiones A (**1**) and B (**2**), one new diphenyl ketone riboside, pestalotione C (**7**), and one new diphenyl ether, pestalotione D (**8**), along with five known compounds isosulochrin dehydrate (**3**), 3,8-dihydroxy-6-methyl-9-oxo-9*H*-xanthene-1-carboxylate (**4**), isosulochrin (**5**), chloroisosulochrin (**6**), and pestalotether D (**9**), were isolated from the crude extract of the plant endophytic fungus *Pestalotiopsis theae* (N635). The structures of the new compounds were unambiguously deduced by HRESIMS and 1D/2D-NMR spectroscopic data. Compound **6** showed modest cytotoxicity against the HeLa cell line with an IC_50_ value of 35.2 μM. Compound **9** also showed cytotoxic to the HeLa and MCF-7 cell lines, with IC_50_ values of 60.8 and 22.6 μM, respectively. Additionally, compounds **1** and **2** exhibited antioxidant activity in scavenging DPPH radical with IC_50_ values of 54.2 and 59.2 μg/mL, respectively.

## 1. Introduction

**Figure 1:**
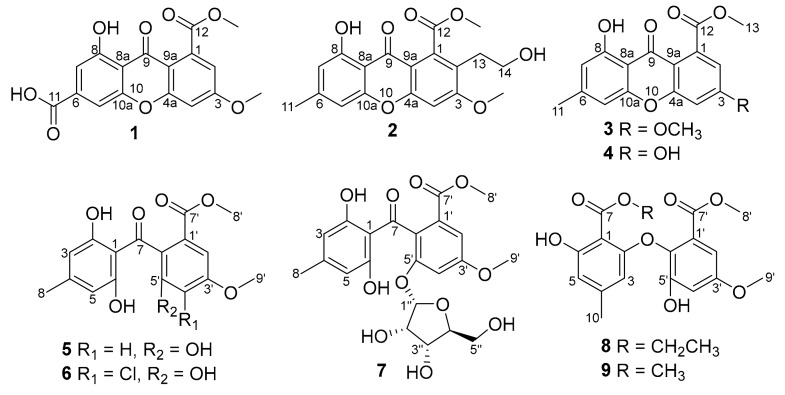
Chemical structures of compounds **1**–**9**.

Fungi are capable of producing a variety of bioactive secondary metabolites [1,2,3]. Endophytic fungi inhabiting the normal tissues of healthy plants have attracted considerable attention due to their ecological and biotechnological potential [4,5]. Special environments and selective pressures have an influence on the metabolic process of endophytes, leading to their enormous biological diversity and a variety of biosynthetic capabilities [6,7,8,9]. The widely distributed endophytic fungi, *Pestalotiopsis* spp., has attracted much attention owing to the discovery of structurally diverse and biologically active secondary metabolites [10,11,12,13,14,15], including the anticancer drug, paclitaxel, which was isolated from *P. guepinii* and *P. microspora* [16,17]. Chemical investigations of the fungus *P. theae* have also yielded bioactive compounds such as cytosporins, phytotoxins, pestalotheols, pestalazines, and pestalamides [18,19,20,21]. In a search for new bioactive natural products from this fungal genus, a strain of *P. theae* (N635), isolated from the branches of the tea plant *Camellia sinensis* (Theaceae) in the suburb of Hangzhou, P. R. China, was grown in different solid-substrate fermentation cultures. Chemical studies of the resulting crude extracts had afforded structurally unique compounds showing an antiproliferative effect against the human tumor cell lines HeLa and MCF-7, including two spiroketals chlorotheolides A and B possessing the unique [4,7] methanochromene and dispirotrione skeletons, and their putative biosynthetic precursors [22]. In addition, two new xanthone derivatives, pestalotiones A (**1**) and B (**2**), one new diphenyl ketone riboside, pestalotione C (**7**), and one new diphenyl ether, pestalotione D (**8**), along with five known compounds isosulochrin dehydrate (**3**) [23] 3,8-dihydroxy-6-methyl-9-oxo-9*H*-xanthene-1-carboxylate (**4**) [24], isosulochrin (**5**) [23], chloroisosulochrin (**6**) [23], and pestalotether D (**9**) [25] (Figure 1), were isolated from the crude extract. All compounds were evaluated for cytotoxicity against a panel of four human tumor cell lines that are commonly used in our laboratory. Meanwhile, their antioxidant activities were also evaluated. Details of the isolation, structure elucidation, biological activities and proposed biosynthetic pathway of these metabolites are reported herein.

## 2. Results and Discussion

### 2.1. Isolation and Structure Elucidation

**Figure 2:**
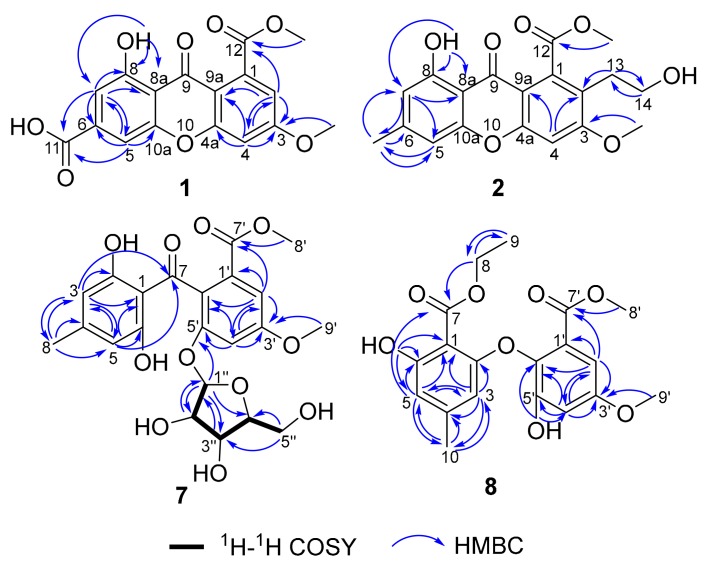
HMBC correlations of compounds **1**, **2**, **7**, and **8** and selected ^1^H–^1^H COSY of compound **7.**

Pestalotione A (**1**) was assigned the molecular formula C_17_H_12_O_8_ (12 degrees of unsaturation) on the basis of HRESIMS data. The UV spectrum of the yellow powder showed four maxima (234, 254, 310, and 369 nm), suggesting a xanthone chromophore [24]. Analysis of its NMR data (Table 1) revealed the presence of one exchangeable proton (*δ*_H_ 12.3), two methoxy groups, 12 aromatic carbons (the carbon C-6 signal resonated at *δ*_C_ 139.4 was determined by HMBC correlations (Figure 2) from H-5 and H-7 to C-6), including four protonated, two carboxylic carbons (*δ*_C_ 165.8, 167.9), and one conjugated ketone carbon (*δ*_C_ 179.5). These data accounted for all the NMR resonances of **1** except for one unobserved exchangeable proton and required **1** to be a tricyclic compound. A detailed NMR data comparison with xanthone derivative isosulochrin dehydrate (**3**) revealed the similarity of them. The ^1^H–^1^H coupling patterns for the four aromatic protons also revealed two *m*-substituted aryl rings. HMBC correlations (Figure 2) from H-2 to C-3, C-4, C-9a, and the carboxylic carbon C-12 (*δ*_C_ 167.9), H-4 to C-2, C-3, C-4a, the ketone carbon C-9 (*δ*_C_ 179.5) and C-9a, H-5 to C-6, C-7, C-9, C-10a, and the carboxylic carbon C-11 (*δ*_C_ 165.8), and from H-7 to C-5, C-6, C-8, C-8a, C-9, and C-11 permitted completion of the xanthone core structure with two carboxylic carbons C-11 and C-12 located at C-6 and C-1, respectively. The cross-peaks from the phenolic proton OH-8 (*δ*_H_ 12.3) to C-7, C-8, and C-8a led to the attachment of the hydroxy groups to C-8. HMBC correlations from two methoxy groups to C-3 and C-12, established the locations of these methoxy groups. The remaining one exchangeable proton was located at C-11 by default. Therefore, the planar structure of compound **1** was established as 1-hydroxy-6-methoxy-8-(methoxycarbonyl)-9-oxo-9*H*-xanthene-3-carboxylic acid, named pestalotione A (Figure 1).

The molecular formula of pestalotione B (**2**) was established as C_19_H_18_O_7_ (11 degrees of unsaturation) on the basis of the HRESIMS. The ^1^H and ^13^C-NMR spectrum (Table 1) of **2** exhibited one exchangeable proton at *δ*_H_ 12.3, two oxygenated methyls, one methyl, two methylenes (one oxygenated), twelve olefinic/aromatic carbons (three of which were protonated), one carboxylic carbon (*δ*_C_ 168.4), and one conjugated ketone carbon (*δ*_C_ 180.3). These data accounted for all the resonances observed in the NMR spectra of **2** except for one unobserved exchangeable proton. The ^1^H- and ^13^C-NMR spectra of **2** displayed signals for structural features similar to **1**, except that the aromatic proton H-2 (*δ*_H_ 7.13) and the carboxylic carbon (*δ*_C_ 165.8) in **1** were replaced by the 2-hydroxyethyl unit (*δ*_H_ 2.87, 3.00, 3.50, 3.56, *δ*_C_ 38.0, 61.5, respectively) and a methyl group (*δ*_H/C_ 2.43/22.4), respectively. This was further confirmed by HMBC correlations (Figure 2) from H_3_-11 to C-5, C-6, and C-7, H_2_-13 to C-2 and C-14, and from H_2_-14 to C-13. Accordingly, compound **2**, namely pestalotione B, was identified as methyl 8-hydroxy-2-(2-hydroxyethyl)-3-methoxy-6-methyl-9-oxo-9*H*-xanthene-1-carboxylate.

The molecular formula of pestalotione C (**7**) was established as C_22_H_24_O_11_ (11 degrees of unsaturation) on the basis of HRESIMS data. Its ^1^H, ^13^C, and HSQC NMR spectroscopic data (Table 2) showed resonances for two exchangeable protons (*δ*_H_ 10.9 and 3.3, respectively), three methyl groups with two oxygenated, one methylene unit, four oxymethines, twelve olefinic/aromatic carbons including four protonated, one carboxylic carbon (*δ*_C_ 167.7), and one conjugated ketone carbon (*δ*_C_ 201.1). Interpretation of these data revealed structural features similar to those presented in isosulochrin (**5**) [23] except for the presence of a furanose unit. Interpretation of the ^1^H–^1^H COSY NMR data (Figure 2) led to the identification of one isolated proton spin-system corresponding to the C-1′′-C-2′′-C-3′′-C-4′′-C-5′′ subunit of structure **7**. The ribose residue was confirmed by comparing the ^13^C-NMR data with those of several furanoside, such as isotorachrysone-6-*O*-α-d-ribofuranoside, and asperflavin ribofuranoside [26,27]. The sugar moiety was further determined as α-form by comparison of the coupling constant (*J*_1__′′__2__′′_ = 4.4 Hz) of the anomeric proton with those of the methyl-α-d-ribofuranoside (*J*_1,2_ = 4.3 Hz) and methyl-β-d-ribofuranoside (*J*_1,2_ = 1.2 Hz) [28]. The key HMBC correlations (Figure 2) from the anomeric proton H-1′′ to C-4′′ (*δ*_C_ 88.1) and C-5′ (*δ*_C_ 155.7) determined the ribose moiety, which was linked to C-5′ through oxygen bond. Upon acid hydrolysis of **7** with methanol/HCl, the liberated sugar was identified as d-ribose by measurement of its specific rotation (${\left[\alpha \right]}_{D}^{25}$−16.0, *c* 0.2, H_2_O) [29]. Thus, compound **7** was elucidated as isosulochrin-5′-*O*-α-d-ribofuranoside, named pestalotione C (**7**).

The molecular formula of pestalotione D (**8**) was established as C_19_H_20_O_8_ (10 degrees of unsaturation) on the basis of HRESIMS data. The overall appearance of ^1^H and ^13^C-NMR spectra (Table 3) of **8** are highly similar to those of **9** except that signals for the methoxyl group (CH_3_O-7) were replaced by those for the ethoxyl group (*δ*_H_/*δ*_C_ 4.47/62.4, 1.40/14.3) in the spectra of **8**, which were supported by the HMBC correlations (Figure 2) from H_2_-8 to C-7 and C-9 and from H_3_-9 to C-8. The chemical structure of compound **8** was elucidated as methyl 2-(2-(ethoxycarbonyl)-3-hydroxy-5-methylphenoxy)-3-hydroxy-5-methoxybenzoate, named pestalotione D (**8**). 

**Figure 3:**
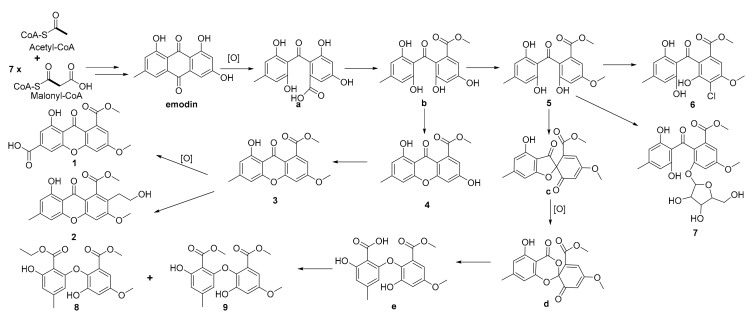
Proposed biosynthetic pathways of compounds **1**–**9**.

Biogenetically, emodin, biosynthesized from one molecule of acetyl-CoA and seven molecules of malonyl-CoA [30], could be the biosynthetic precursor not only for compounds **1**–**4**, but also for **5**–**9**, first via oxidation and methylation to form the key intermediate **b**, and then followed by a series of reactions through different routes to form **1**–**9**. The proposed precursor and the reaction cascades leading to the generation of these metabolites are illustrated in Figure 3.

### 2.2. Bioactivities

Compounds **1**−**9** were tested for cytotoxicity against a panel of four human tumor cancer cell lines, HeLa (human cervical carcinoma cell line), MCF-7 (human breast cancer cell line), HepG2 (human hepatoma cell line), and ACHN (human renal carcinoma cell line). Compounds **6** and **9** showed cytotoxic to the HeLa cell line, with IC_50_ values of 35.2 and 60.8 μM, respectively, whereas the positive control cisplatin showed IC_50_ values of 15.1 and 5.5 μM, respectively. Compound **9** also showed cytotoxic to the MCF-7 cell line, with IC_50_ value of 22.6 μM, whereas the positive control cisplatin showed an IC_50_ value of 5.8 μM. Other compounds did not show detectable inhibitory effects on the cell lines tested at 100 μM. Meanwhile, their antioxidant activity was also evaluated by the DPPH (2,2-diphenyl-1-picrylhydrazyl radical) scavenging method with ascorbic acid as positive control (IC_50_ = 6.0 μg/mL). Only compounds **1** and **2** exhibited weak DPPH scavenging activity with respective IC_50_ values of 54.2 and 59.2 μg/mL. 

Xanthone derivatives were found to display diverse activities, such as tumor cytotoxic activity, antivirus, antibacterial, antifungal, and antimalaria activities [31,32,33,34]. *Pestalotiopsis* sp. was an interesting producer of bioactive metabolites. In our previous study, two spiroketals chlorotheolides A and B from *P*. *theae* showed an antiproliferative effect against the human tumor cell lines HeLa and MCF-7 [22]. In the current study, new structural metabolites with cytotoxic and antioxidant activities were identified from the same fungus. This highlighted the high potential of bioprospecting larvicides from the endophytic fungi.

## 3. Experimental Section 

### 3.1. General Experimental Procedures

Optical rotations were measured on an Anton Paar MCP 200 Automatic Polarimeter and UV data were obtained on a Thermo Genesys-10S UV/Vis spectrophotometer. IR data were recorded using a Nicolet IS5 FT-IR spectrophotometer. ^1^H and ^13^C-NMR data were acquired with Bruker Avance-400 and -500 spectrometer using solvent signals (acetone-*d*_6_: *δ*_H_ 2.05/*δ*_C_ 29.8, 206.3; DMSO-*d*_6_: *δ*_H_ 2.50/*δ*_C_ 39.5; methanol-*d*_4_: *δ*_H_ 3.31/*δ*_C_ 49.0; CDCl_3_: *δ*_H_ 7.26/*δ*_C_ 77.2 ppm) as references. The HSQC and HMBC experiments were optimized for 145.0 and 8.0 Hz, respectively. ESIMS and HRESIMS data were obtained using an Agilent Accurate-Mass-Q-TOF LC/MS 6520 instrument equipped with an electrospray ionization (ESI) source. The fragmentor and capillary voltages were kept at 125 and 3500 V, respectively. Nitrogen was supplied as the nebulizing and drying gas. The temperature of the drying gas was set at 300 °C. The flow rate of the drying gas and the pressure of the nebulizer were 10 L/min and 10 psi, respectively. All MS experiments were performed in positive ion mode. Full-scan spectra were acquired over a scan range of *m/z* 100–1000 at 1.03 spectra/s. HPLC separations were performed on an Agilent 1260 instrument equipped with a variable-wavelength UV detector.

### 3.2. Fungal Material

The culture of *P. theae* (N635) was isolated from *Camellia sinensis* (Theaceae) in Hangzhou, People’s Republic of China. The isolate was identified based on sequence analysis of the ITS region of the rDNA (GenBank Accession No. KF641183). Firstly, the strain was cultured on potato dextrose agar (PDA) at 25 °C for 10 days. Secondly, agar plugs were cut into small pieces (about 0.5 × 0.5 × 0.5 cm^3^) under aseptic conditions, and every five pieces were inoculated into an Erlenmeyer flask (250 mL) containing 50 mL of media (0.4% glucose, 1% malt extract, and 0.4% yeast extract) with final pH 6.5. The flasks inoculated with the media were used as seed cultures after incubating at 25 °C on a rotary shaker at 170 rpm for 5 days. Spore inoculum was prepared by suspension in sterile, distilled H_2_O, resulting in a final spore/cell suspension of 1 × 10^6^/mL. Thirdly, each Fernbach flask (500 mL) containing 80 g of rice and 120 mL of distilled H_2_O was then sealed, soaked overnight, and autoclaved at 15 psi for 30 min. After cooling to room temperature, 5.0 mL of the spore inoculum obtained from liquid phase cultivation was added to each flask and incubated at 25 °C for 40 days.

### 3.3. Extraction and Isolation

The fermented rice material was extracted several times with EtOAc (4 × 4.0 L), and the organic solvent was evaporated to dryness by vacuum steamer to obtain the crude extract (15 g), which was fractionated by silica gel vacuum liquid chromatography (VLC) using petroleum ether−EtOAc gradient elution. The fraction (1.5 g) eluted with 35%–55% EtOAc were combined and separated by ODS CC using MeOH–H_2_O gradient elution. A 230 mg subfraction eluted with 60% MeOH was separated by Sephadex LH-20 CC eluting with MeOH, and the resulting subfraction were purified by RP HPLC (Agilent Zorbax SB-C_18_ column, 5 μm; 9.4 × 250 mm, 65%–75% MeOH in H_2_O for 30 min, 2.0 mL/min) to afford **1** (2.1 mg, *t*_R_ 20.82 min) and **2** (2.0 mg, *t*_R_ 26.0 min). The remaining subfractions eluted with 40%, 50%, and 80% MeOH were separated by Sephadex LH-20 CC eluting with MeOH, and the resulting subfractions were purified by RP HPLC to afford **3** (4.6 mg, *t*_R_ 32.51 min; 65%–78% MeOH in H_2_O for 35 min, 2.0 mL/min), **4** (3.5 mg, *t*_R_ 21.02 min, 60%–80% MeOH in H_2_O for 35 min, 2.0 mL/min), **5** (7.2 mg, *t*_R_ 12.32 min, 50%–68% MeOH in H_2_O for 15 min, 2.0 mL/min), **6** (6.3 mg, *t*_R_ 23.12 min, 60%–90% MeOH in H_2_O for 30 min, 2.0 mL/min). The fraction (236 mg) eluted with 90% EtOAc was combined and separated by Sephadex LH-20 using CH_2_Cl_2_–MeOH = 1:1 gradient elution. The resulting subfraction was purified by RP HPLC to afford **7** (10.0 mg, *t*_R_ 26.5 min, 43% MeOH in H_2_O for 30 min, 2.0 mL/min). The fraction (2.7 g) eluted with 15% EtOAc was combined and separated by a normal pressure columnar using petroleum ether−EtOAc gradient elution. The resulting subfraction (1.0 g) was separated by Sephadex LH-20 CC eluting with CH_2_Cl_2_–MeOH = 1:1 gradient elution, and was purified by RP HPLC to afford **8** (1.5 mg, *t*_R_ 36.1 min, 59% MeOH in H_2_O for 40 min, 2.0 mL/min). The fraction (4.7 g) eluted with 20% EtOAc was combined and separated by normal pressure columnar using petroleum ether−EtOAc gradient elution. The resulting subfraction (0.8 g) was separated by medium pressure column eluting with 55%–95% MeOH gradient elution for 80 min, 10 mL/min, and the fraction collected for 40–43 min was purified by RP HPLC to afford **9** (1.5 mg, *t*_R_ 34.0 min, 56% MeOH in H_2_O for 50 min, 2.0 mL/min).

### 3.4. Spectroscopic Data (uv and IR, ms)

*Pestalotione A* (**1**): yellow powder; UV(MeOH) λ_max_ (log ε) 234 (3.8), 254 (3.6), 310 (3.5), 369 (3.1) nm; IR (neat) ν_max_ 3091, 2947, 1734, 1697, 1648, 1602, 1562, 1414, 1322, 1252, 1146, 1038, 1013 and 771 cm^−1^; ^1^H-NMR, ^13^C-NMR, and HMBC data see Table 1 (see Supplementary Materials); HRESIMS m/z 345.0623 (calcd for C_17_H_13_O_8_, 345.0605).

*Pestalotione B* (**2**): yellow powder; UV(MeOH) λ_max_ (log ε) 242 (4.2), 271 (4.3), 302 (3.9), 360 (3.6) nm; IR (neat) ν_max_ 3503, 2948, 1731, 1652, 1614, 1588, 1488, 1422, 1368, 1264, 1213, 1030 cm^−1^; ^1^H-NMR, ^13^C-NMR, and HMBC data see Table 1; HRESIMS m/z 359.1121 (calcd for C_19_H_19_O_7_, 359.1125).

*Pestalotione C* (**7**): yellow oil; ${\left[\alpha \right]}_{D}^{25}$+25.0 (c 0.1, MeOH); UV (MeOH) λ_max_ (log ε) 214 (3.0), 286 (1.7) nm; IR (neat) ν_max_ 3423, 2952, 2846, 1721, 1635, 1607, 1445, 1326, 1250, 1045, 1025, 830, 791 cm^–1^; ^1^H-NMR, ^13^C-NMR and HMBC data see Table 2; HRESIMS m/z 487.1217 (calcd for C_22_H_24_O_11_ Na, 487.1216).

*Hydrolysis of pestalotione C* (**7**): The compound **7** (1 mg) was dissolved in acetone (300 μL) and added to 700 μL 6 M HCl. After hydrolysis at 100 °C for 48 h, and adding double distilled water (3 mL) into the reaction bulb, the aglycone was extracted with CH_2_Cl_2_ (3 × 10 mL). The aqueous was rotary evaporate-dried, dissolved in water (1 mL), then the specific rotation was measured. The rotation recorded for the ribose isolated was ${\left[\alpha \right]}_{D}^{25}$−16.0 (c 0.2, H_2_O), which closely matched that for the d-ribose ${\left[\alpha \right]}_{D}^{25}$−23.0 (c 0.02, H_2_O) [35]. 

### 3.5. MTS Assay

The assay plate was read at 490 nm using a microplate reader. The assay was run in triplicate. In a 96-well plate, each well was plated with (2–5) × 10^3^ cells (depending on the cell multiplication rate). After cell attachment overnight, the medium was removed, and each well was treated with 100 µL of medium containing 0.1% DMSO, or appropriate concentrations of the test compounds and the positive control cisplatin (100 mM as stock solution of a compound in DMSO and serial dilutions; the test compounds showed good solubility in DMSO and did not precipitate when added to the cells). The plate was incubated at 37 °C for 48 h in a humidified, 5% CO_2_ atmosphere. Proliferation was assessed by adding 20 μL of MTS (Promega) to each well in the dark, followed by incubation at 37 °C for 90 min. The assay plate was read at 490 nm using a microplate reader. The assay was run in triplicate [36].

### 3.6. Antioxidant Assay

The DPPH scavenging assay was performed according to the former reported method [37]. The DPPH radical scavenging test was conducted in a 96-well plate. The tested compounds **1**–**9** were added to 50 µL (0.34 mmol/L) DPPH solution in ethanol solutions at a range of 50 µL solutions of different concentrations (12.5, 25, 50, 100, and 200 μM). After 30 min of incubation at 37 °C in the dark environment, the absorbance was read at 517 nm using a microplate reader, employing distilled water as a blank for baseline correction. The data that represent three independent experiments was calculated, and ascorbic acid was used as a positive control.

## 4. Conclusions

In summary, nine polyketides including four new ones were isolated from the crude extract of the fungus *P. theae*. Compounds **1** and **2** exhibited antioxidant activity, while compounds **6** and **9** showed moderate cytotoxic to the human tumor cells. The discovery of these secondary metabolites further expanded the structural diversity of the natural products produced by the fungal genus *Pestalotiopsis*.

## Figures and Tables

**Table 1:** NMR data for compounds **1** (DMSO-*d*_6_) and **2** (acetone-*d*_6_).

Pos.	1			2		
	*δ*_H_*^a^* (*J* in Hz)	*δ*_C_ *^b^*	HMBC*^c^*	*δ*_H_*^a^* (*J* in Hz)	*δ*_C_ *^b^*	HMBC*^c^*
1		134.7, qC			141.2, qC	
2	7.13, d (2.2)	112.9, CH	3, 4, 9a, 12		118.5, qC	
3		165.3, qC			166.7, qC	
4	7.30, d (2.2)	101.9, CH	2, 3, 4a, 9, 9a	7.22, s	101.3, CH	2, 3, 4a, 9, 9a
4a		158.1, qC			159.5, qC	
5	7.45, s	107.5, CH	6, 7, 9, 10a, 11	6.83, s	108.2, CH	7, 8a, 9, 10a, 11
6		139.4, qC			150.3, qC	
7	7.23, s	110.6, CH	5, 6, 8, 8a, 9, 11	6.63, s	112.4, CH	5, 8, 8a, 11,
8		160.7, qC			162.3, qC	
8a		110.1, qC			107.1, qC	
9		179.5, qC			180.3, qC	
9a		110.3, qC			111.7, qC	
10a		155.3, qC			156.8, qC	
11		165.8, qC		2.43, s	22.4, CH_3_	5, 6, 7
12		167.9, qC			168.4, qC	
13a				3.00, m	38.0, CH_2_	2, 14
13b				2.87, m		2, 14
14a				3.56, m	61.5, CH_2_	13
14b				3.50, m		13
CH_3_O-3	3.98, s	56.8, CH_3_	3	4.14, s	57.9, CH_3_	3
CH_3_O-12	3.89, s	52.8, CH_3_	12	3.97, s	53.1, CH_3_	12
HO-8	12.3, s		7, 8, 8a	12.3, s		7, 8, 8a

*^a^* Recorded at 500 MHz. *^b^* Recorded at 125 MHz. *^c^*HMBC correlations, optimized for 8 Hz, are from proton(s) stated by the indicated carbon

**Table 2:** NMR data for compound **7** (methanol-*d*_4_).

Pos.	*δ*_H_*^a^* (*J* in Hz)	*δ*_C_ *^b^*	HMBC*^c^*
1		110.9, qC	
2		163.2, qC	
3	6.13, s	109.0, CH	1, 2, 5, 7, 8
4		149.6, qC	
5	6.13, s	109.0, CH	1, 3, 6, 7, 8
6		163.2, qC	
7		201.1, qC	
8	2.20, s	22.1, CH_3_	3, 4, 5
1′		130.6, qC	
2′	7.17, d (2.4)	109.0, CH	1′, 3′, 4′, 6′, 7′
3′		161.6, qC	
4′	7.03, d (2.4)	108.2, CH	2′, 3′, 5′, 6′
5′		155.7, qC	
6′		130.4, qC	
7′		167.7, qC	
8′	3.70, s	52.6, CH_3_	7′
9′	3.86, s	56.2, CH_3_	3′
1′′	5.56, d (4.4)	103.3, CH	5′, 2′′, 3′′, 4′′
2′′	4.00, dd (6.4, 4.4)	73.3, CH	1′′
3′′	3.90, dd (6.4, 3.2)	70.7, CH	1′′
4′′	3.96, dd (6.8, 3.2)	88.1, CH	
5′′	3.55, m	63.0, CH_2_	3′′, 4′′

*^a^* Recorded at 400 MHz. *^b^* Recorded at 100 MHz. *^c^* HMBC correlations, optimized for 8 Hz, are from proton(s) stated by the indicated carbon.

**Table 3:** NMR data for compound **8** (CDCl_3_).

Pos.	*δ*_H_*^a^ (J* in Hz)	*δ*_C_ *^b^*	HMBC*^c^*
1		102.1, qC	
2		158.2, qC	
3	5.94, d (1.2)	107.1, CH	1, 2, 4, 5, 7, 10
4		146.6, qC	
5	6.49, d (1.2)	112.5, CH	1, 3, 6, 7, 10
6		162.1, qC	
7		169.7, qC	
8	4.47, q (7.2)	62.4, CH_2_	7, 9
9	1.40, t (7.2)	14.3, CH_3_	8
10	2.16, s	22.2, CH_3_	3, 4, 5
1′		125.5, qC	
2′	7.05, d (3.2)	107.3, CH	1′, 3′, 4′, 6′, 7′
3′		157.5, qC	
4′	6.81, d (3.2)	107.3, CH	2′, 3′, 5′, 6′
5′		150.7, qC	
6′		135.4, qC	
7′		165.4, qC	
8′	3.74, s	52.5, CH_3_	7′
9′	3.84, s	55.9, CH_3_	3′
OH-6	10.54, s		1, 5, 6
OH-5′	6.84, br s		4′, 6′

*^a^* Recorded at 400 MHz. *^b^* Recorded at 100 MHz. *^c^* HMBC correlations, optimized for 8 Hz, are from proton(s) stated by the indicated carbon.

## Supplementary Materials

Supplementary materials can be accessed at: https://www.mdpi.com/1420-3049/25/3/470/s1.
