# Supplementary material for: Pestalotiones A–D: Four New Secondary Metabolites from the Plant Endophytic Fungus Pestalotiopsis Theae
Source: Molecules. 2020 Jan 22;25(3):470. doi: 10.3390/molecules25030470 (PMC7037426; doi:10.3390/molecules25030470)

Supporting Information

Pestalotiones A–D, four new secondary metabolites from the plant endophytic fungus *Pestalotiopsis theaea*

Longfang Guo [1,2,Δ], Jie Lin [3,Δ], Shubin Niu[4], Shuchun Liu [1], Ling Liu [1,*]

[1] State Key Laboratory of Mycology, Institute of Microbiology, Chinese Academy of Sciences, Beijing 100101, China

[2] University of Chinese Academy of Sciences, Beijing 100039, China

[3] Jiangsu Key Laboratory for Biofunctional Molecules, College of Life Science and Chemistry, Jiangsu Second Normal University, Nanjing, China

[4] School of Biological Medicine, Beijing City University, Beijing 100083, China

* Corresponding author: Tel: 86-10-64806153; E-mail: liul@im.ac.cn

Δ Contributed equally to this work.

Figure S1. ^{1}H NMR spectrum of pestalotione A (**1**; 500 MHz, DMSO-d_6)....................3
Figure S2. ^{13}C NMR spectrum of pestalotione A (**1**; 125 MHz, DMSO-d_6)....................4
Figure S3. HSQC spectrum of pestalotione A (**1**; 500 MHz, DMSO-d_6)....................5
Figure S4. HMBC spectrum of pestalotione A (**1**; 500 MHz, DMSO-d_6)....................6
Figure S5. ^{1}H NMR spectrum of pestalotione B (**2**; 500 MHz, acetone-d_6)....................7
Figure S6. ^{13}C NMR spectrum of pestalotione B (**2**; 125 MHz, acetone-d_6)....................8
Figure S7. HSQC spectrum of pestalotione B (**2**; 500 MHz, acetone-d_6)....................9
Figure S8. HMBC spectrum of pestalotione B (**2**; 500 MHz, acetone-d_6)....................10
Figure S9. ^{1}H NMR spectrum of pestalotione C (**7**; 400 MHz, methanol-d_4)....................11
Figure S10. ^{13}C NMR spectrum of pestalotione C (**7**; 100 MHz, methanol-d_4)....................12
Figure S11. ^{1}H-^{1}H COSY spectrum of pestalotione C (**7**; 400 MHz, methanol-d_4)....................13
Figure S12. HSQC spectrum of pestalotione C (**7**; 400 MHz, methanol-d_4)....................14
Figure S13. HMBC spectrum of pestalotione C (**7**; 400 MHz, methanol-d_4)....................15
Figure S14. ^{1}H NMR spectrum of pestalotione D (**8**; 400 MHz, $CDCl_3$)....................16
Figure S15. ^{13}C NMR spectrum of pestalotione D (**8**; 100 MHz, $CDCl_3$)....................17
Figure S16. HSQC spectrum of pestalotione D (**8**; 400 MHz, $CDCl_3$)....................18
Figure S17. HMBC spectrum of pestalotione D (**8**; 400 MHz, $CDCl_3$)....................19

Figure S1. ^{1}H NMR spectrum of pestalotione A (**1**; 500 MHz, DMSO-*d*6)

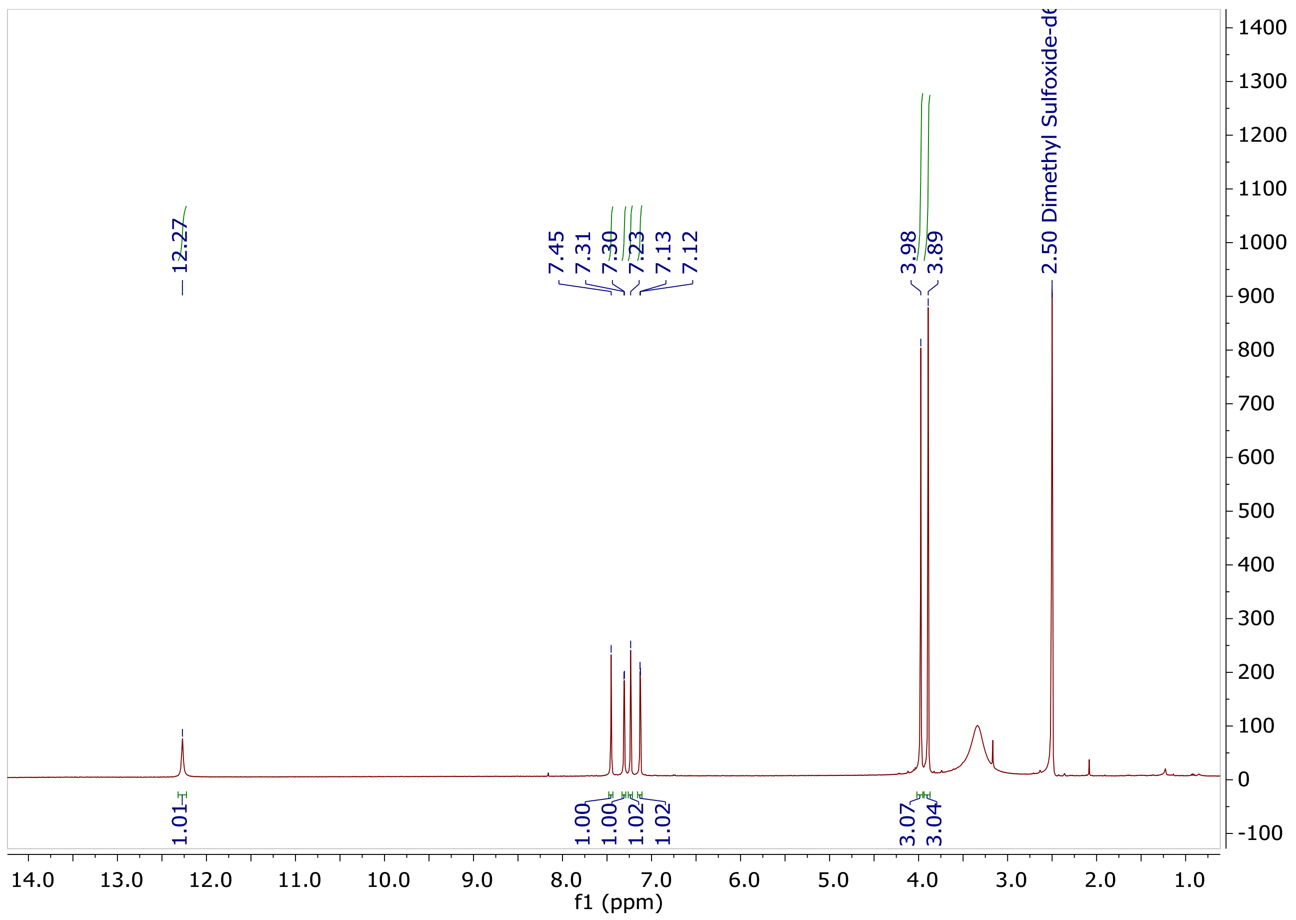

Figure S2. ^{13}C NMR spectrum of pestalotione A (**1**; 125 MHz, DMSO-d_6)

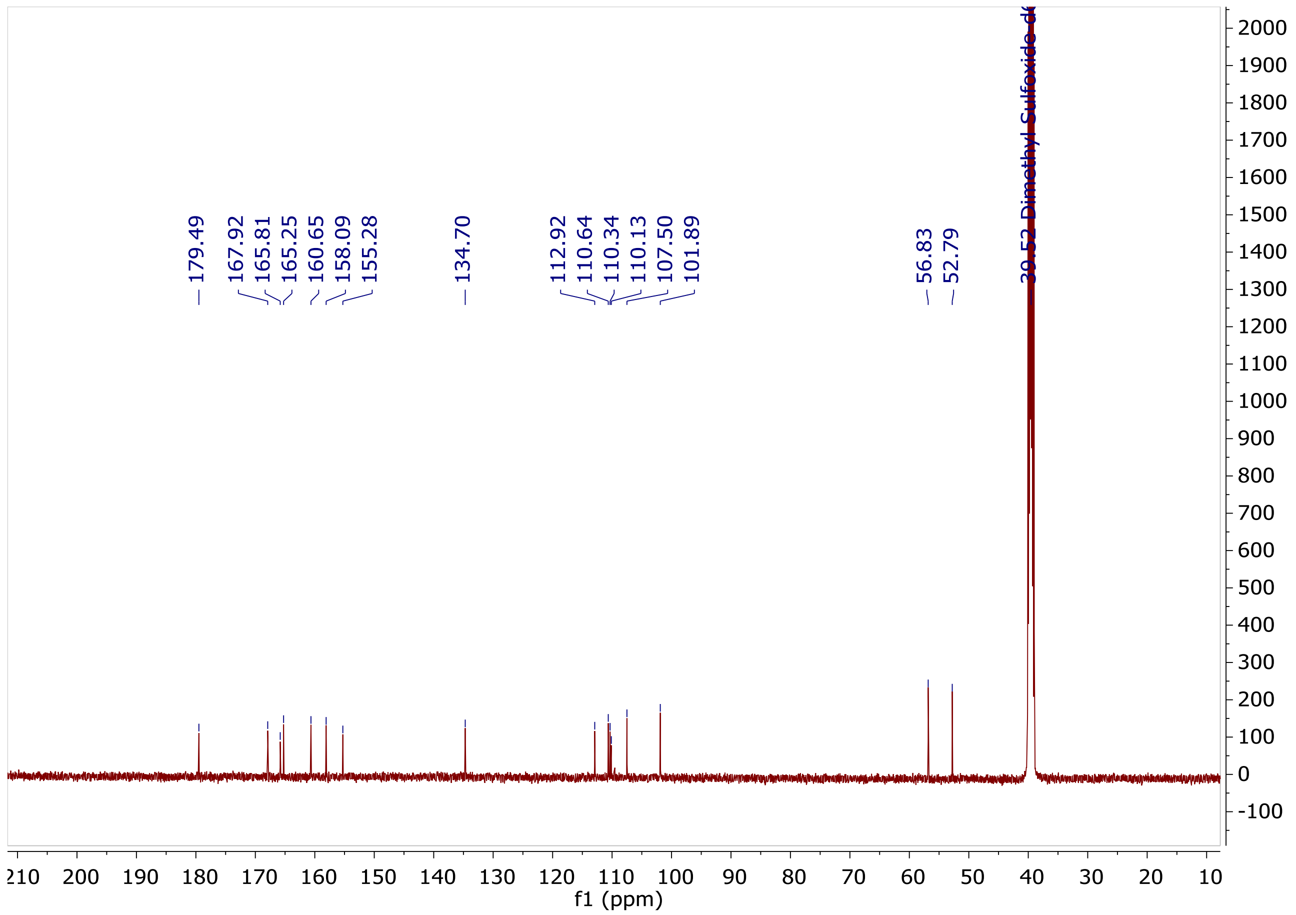

Figure S3. HSQC spectrum of pestalotione A (**1**; 500 MHz, DMSO-d_6)

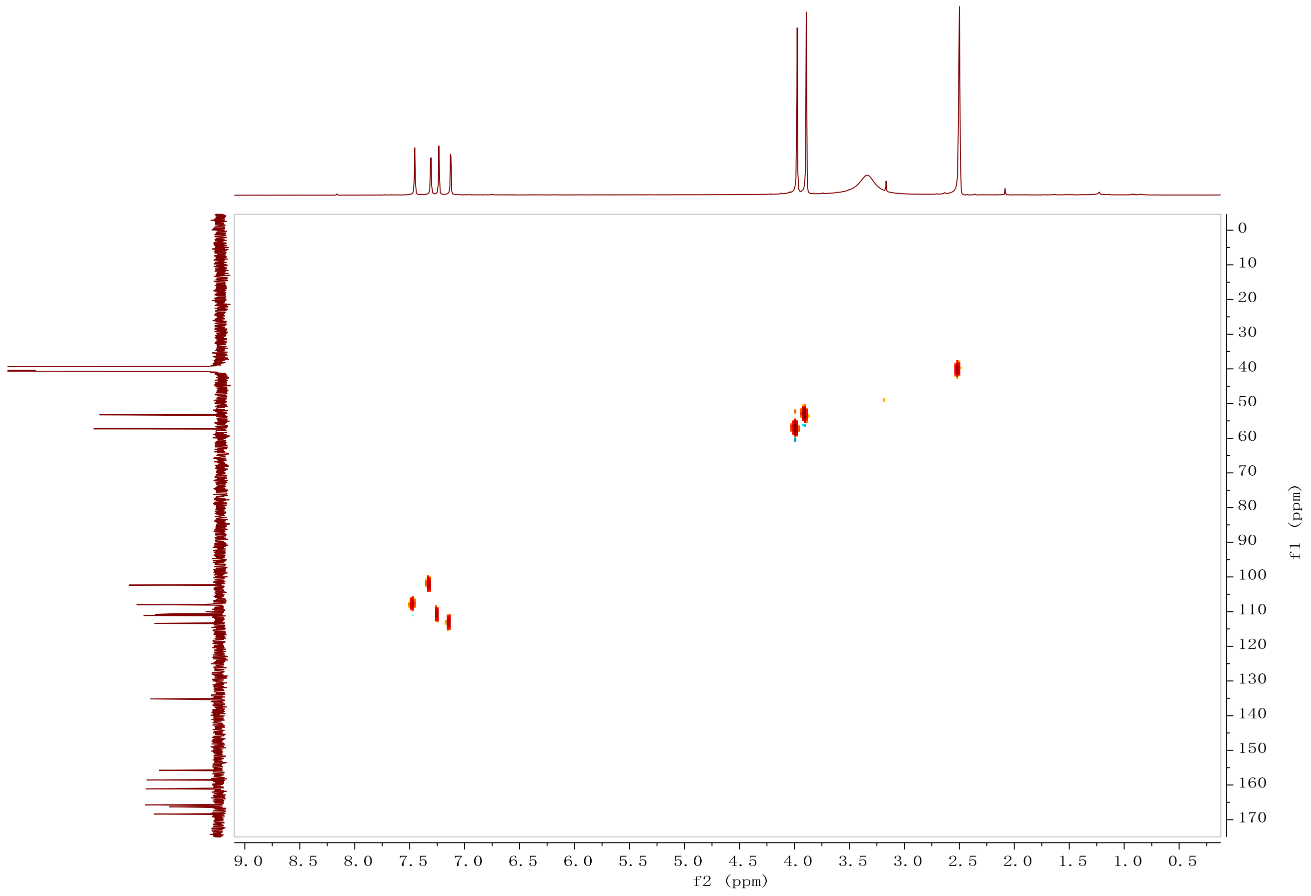

Figure S4. HMBC spectrum of pestalotione A (**1**; 500 MHz, DMSO-d_6)

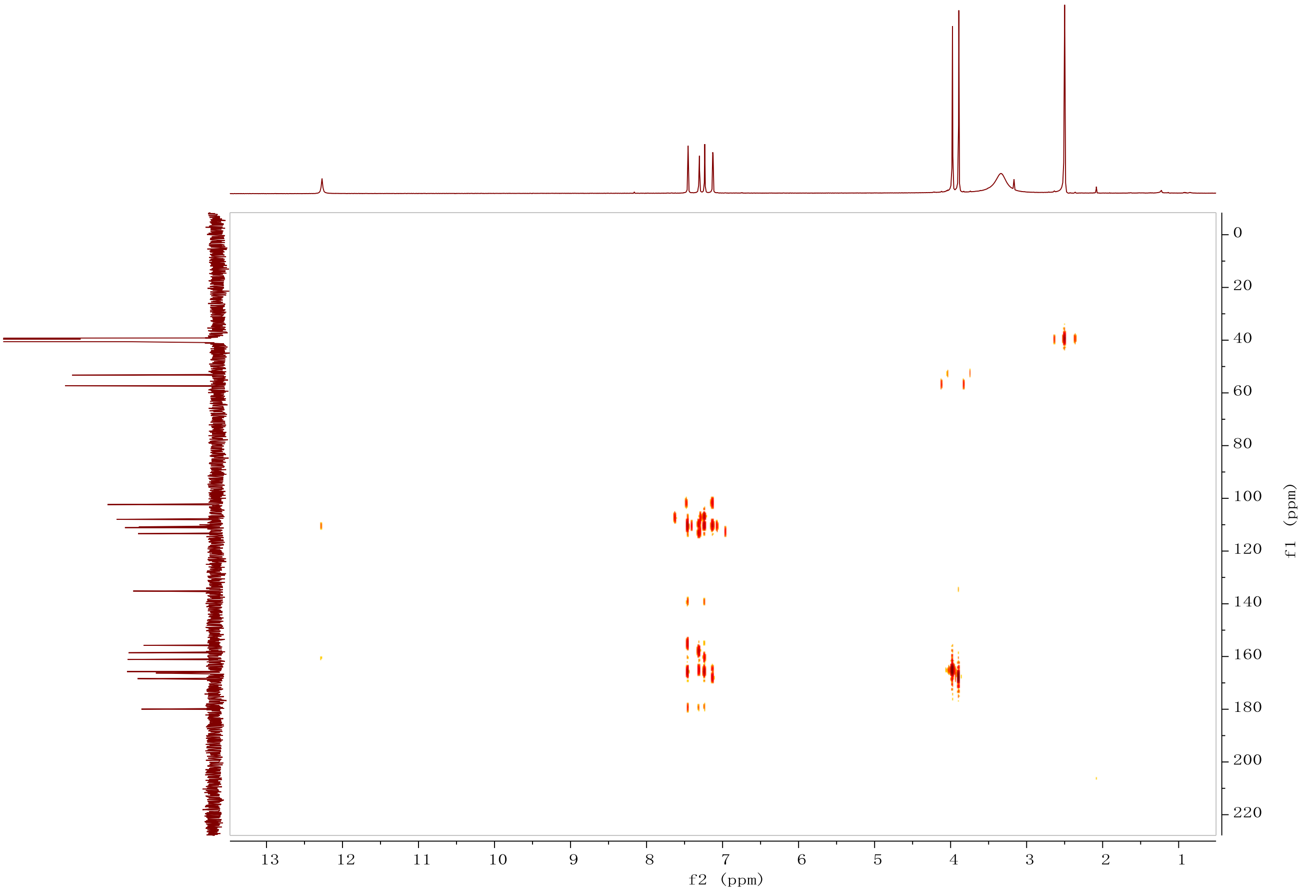

Figure S5. ^{1}H NMR spectrum of pestalotione B (**2**; 500 MHz, acetone-d_6)

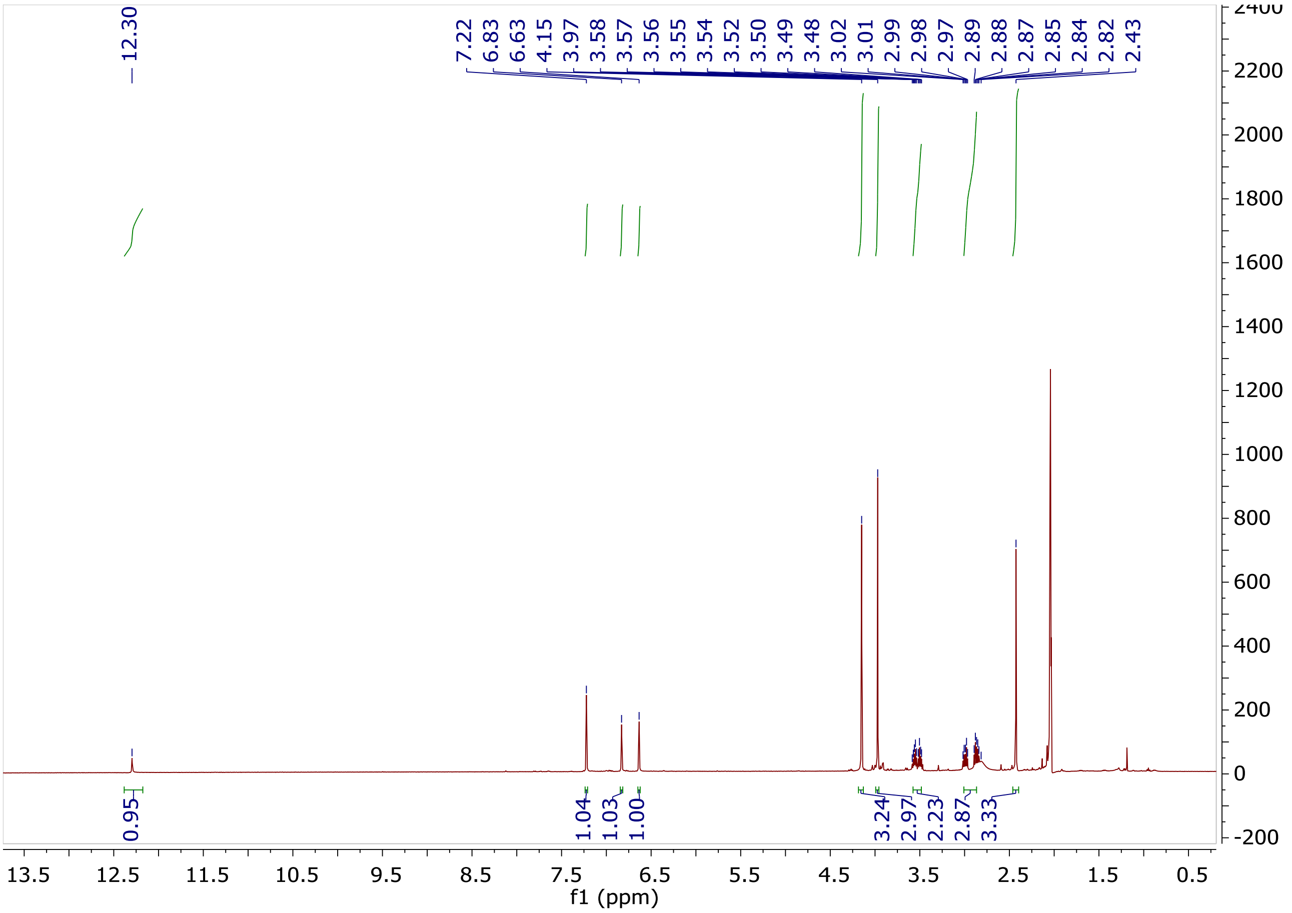

Figure S6. ^{13}C NMR spectrum of pestalotione B (**2**; 125 MHz, acetone-d_6)

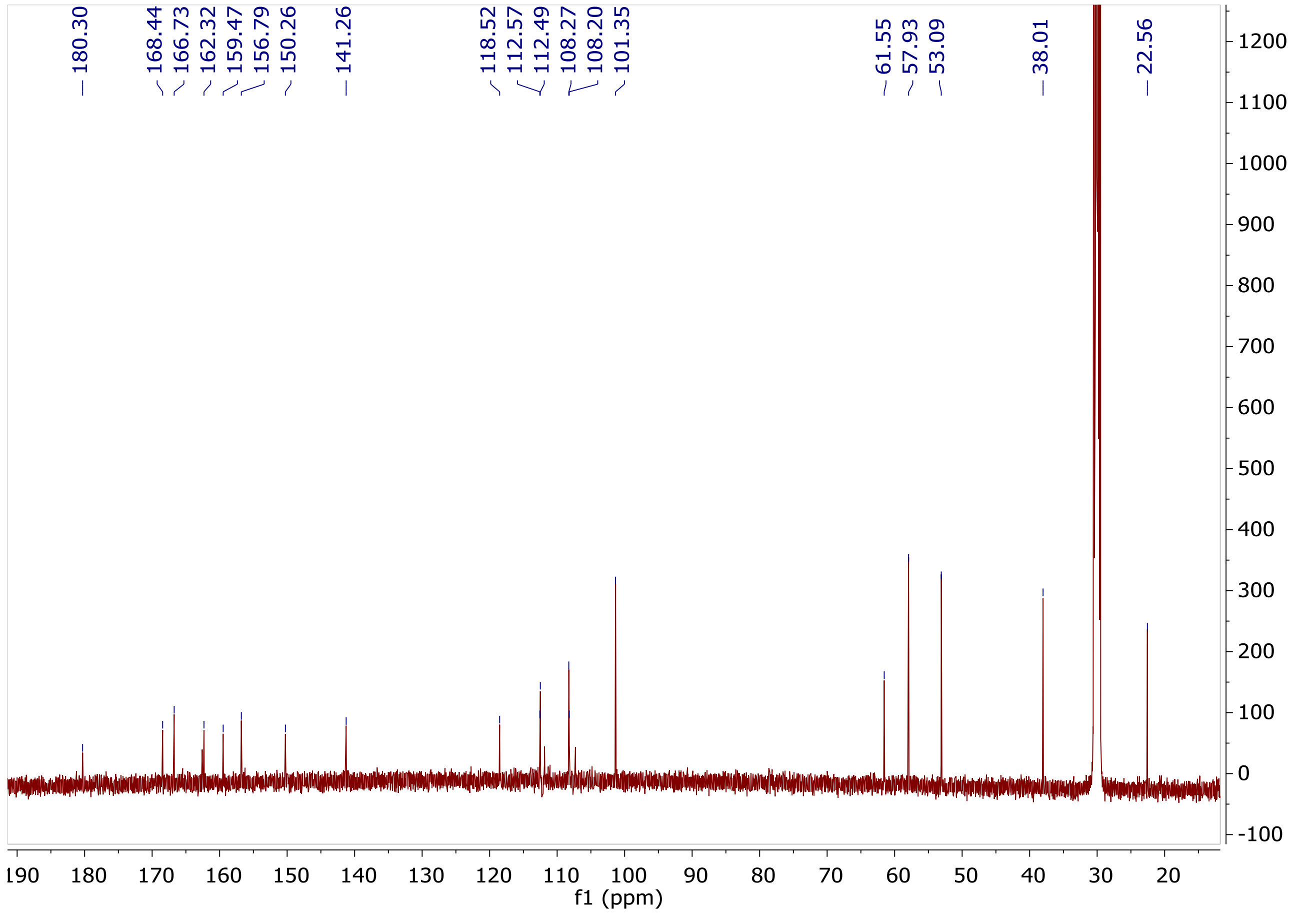

Figure S7. HSQC spectrum of pestalotione B (**2**; 500 MHz, acetone-d_6)

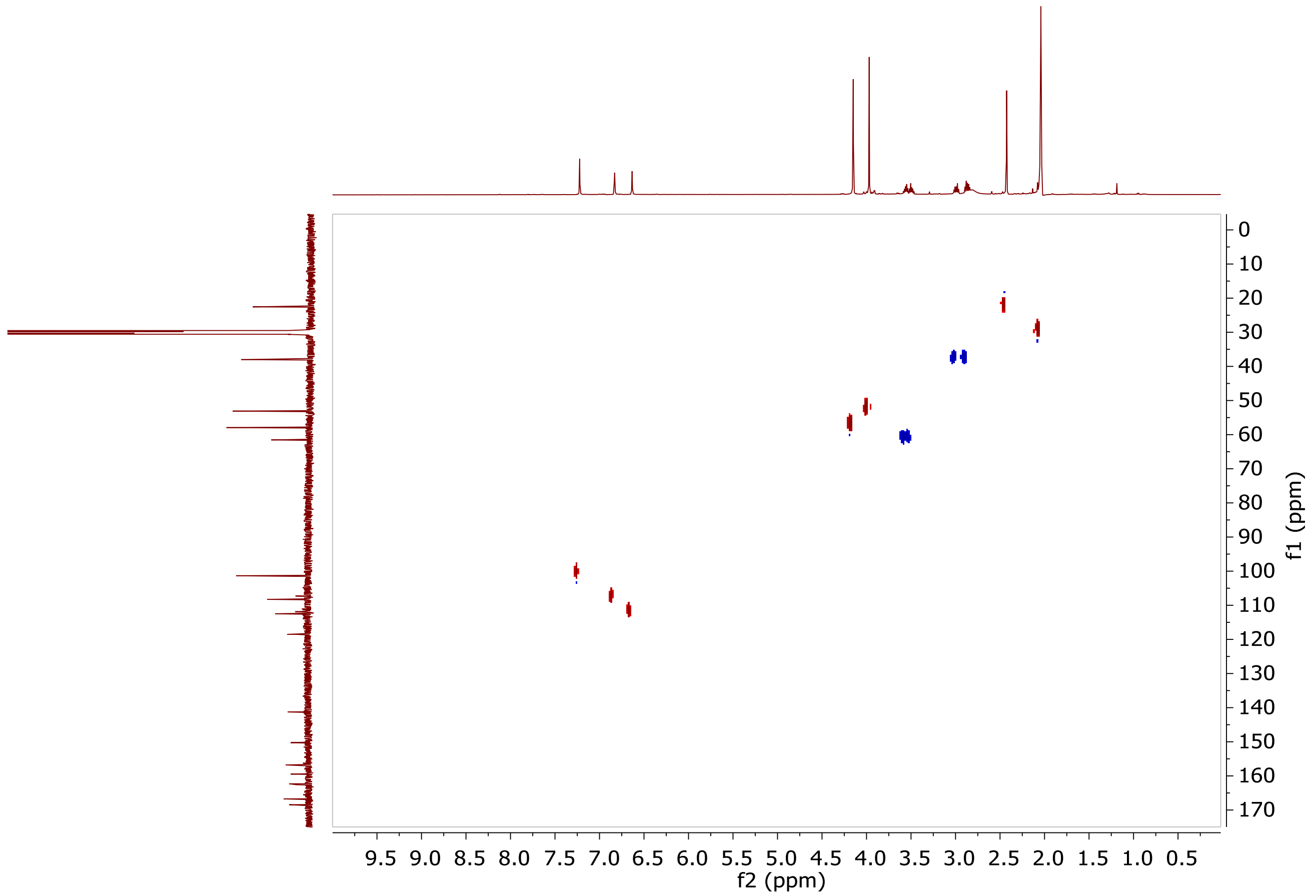

Figure S8. HMBC spectrum of pestalotione B (**2**; 500 MHz, acetone-d_6)

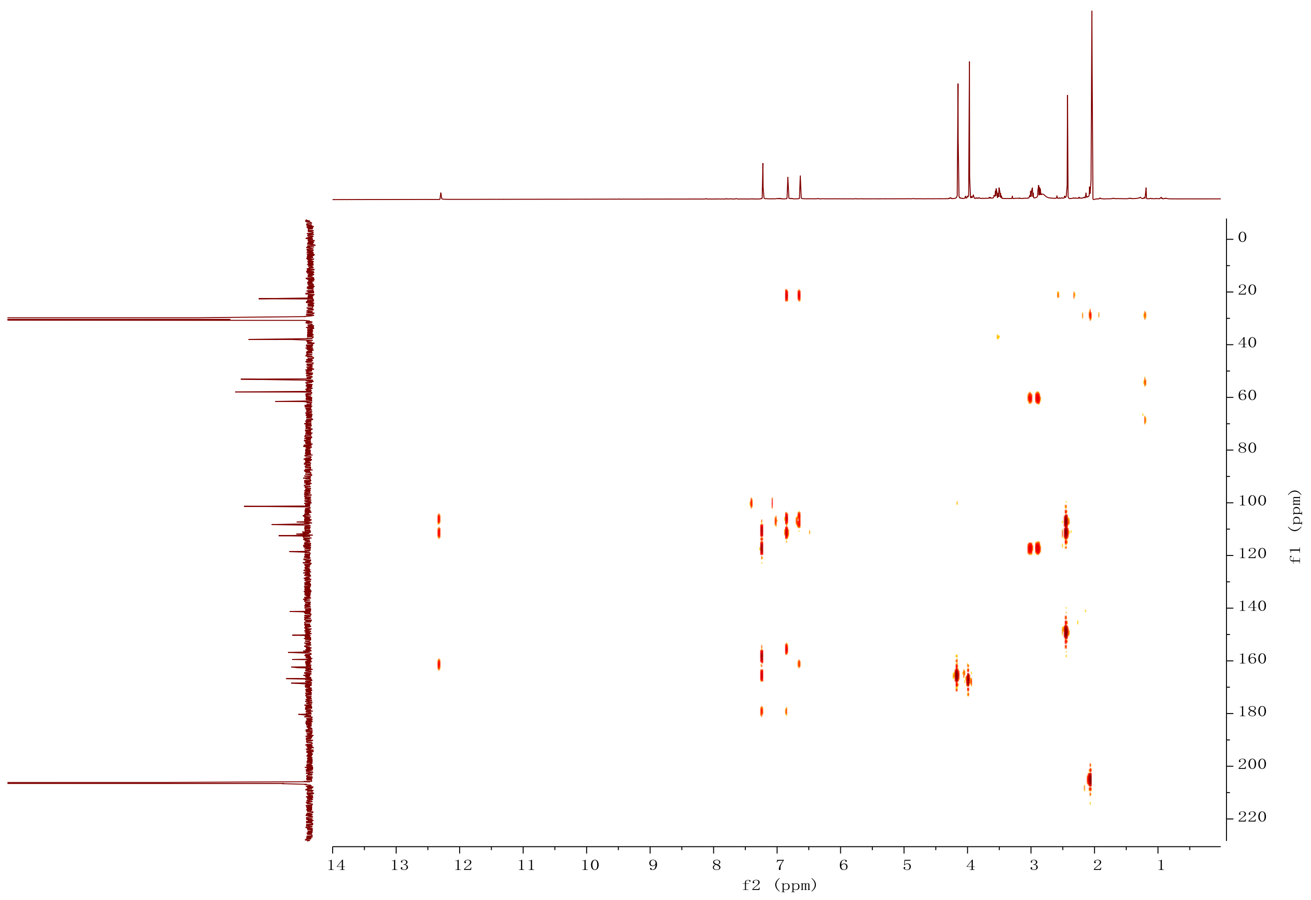

Fig. S9 ^{1}H NMR spectrum of pestalotione C (**7**; 400 MHz, methanol-d_4)

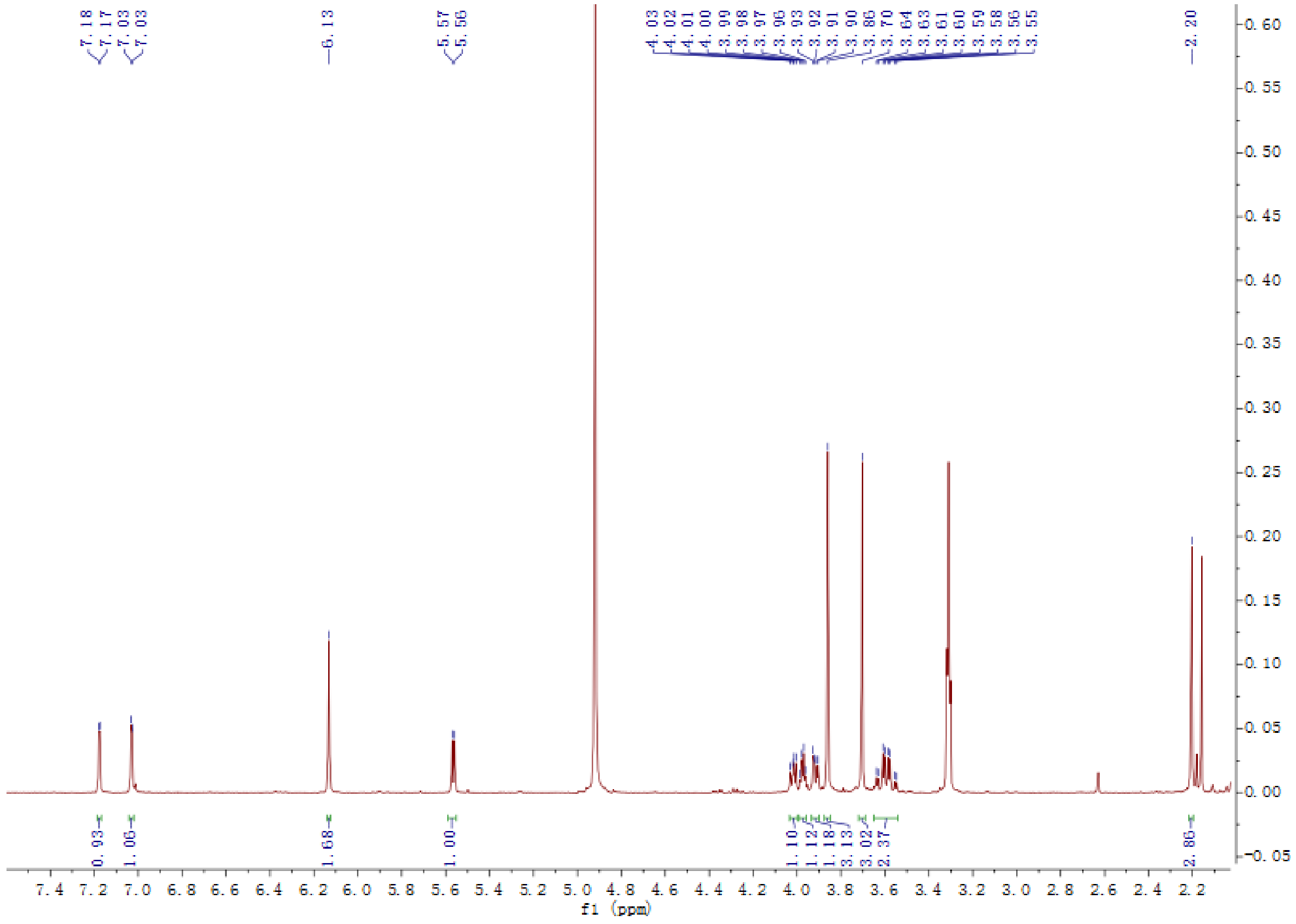

Fig. S10 ^{13}C NMR spectrum of pestalotione C (**7**; 100 MHz, methanol-d_4)

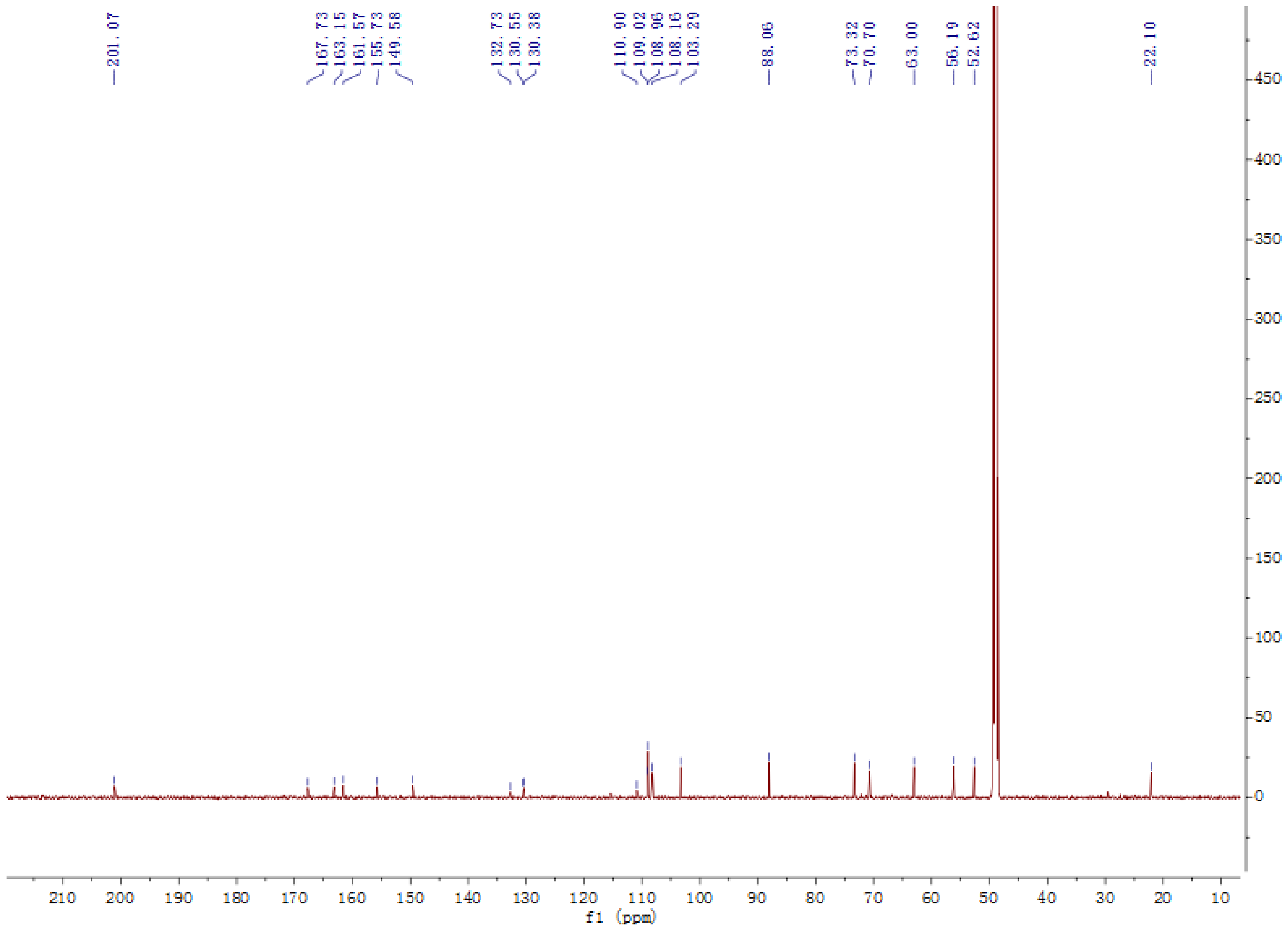

Fig. S11 ^{1}H-^{1}H COSY spectrum of pestalotione C (**7**; 400 MHz, methanol-*d*$_4$)

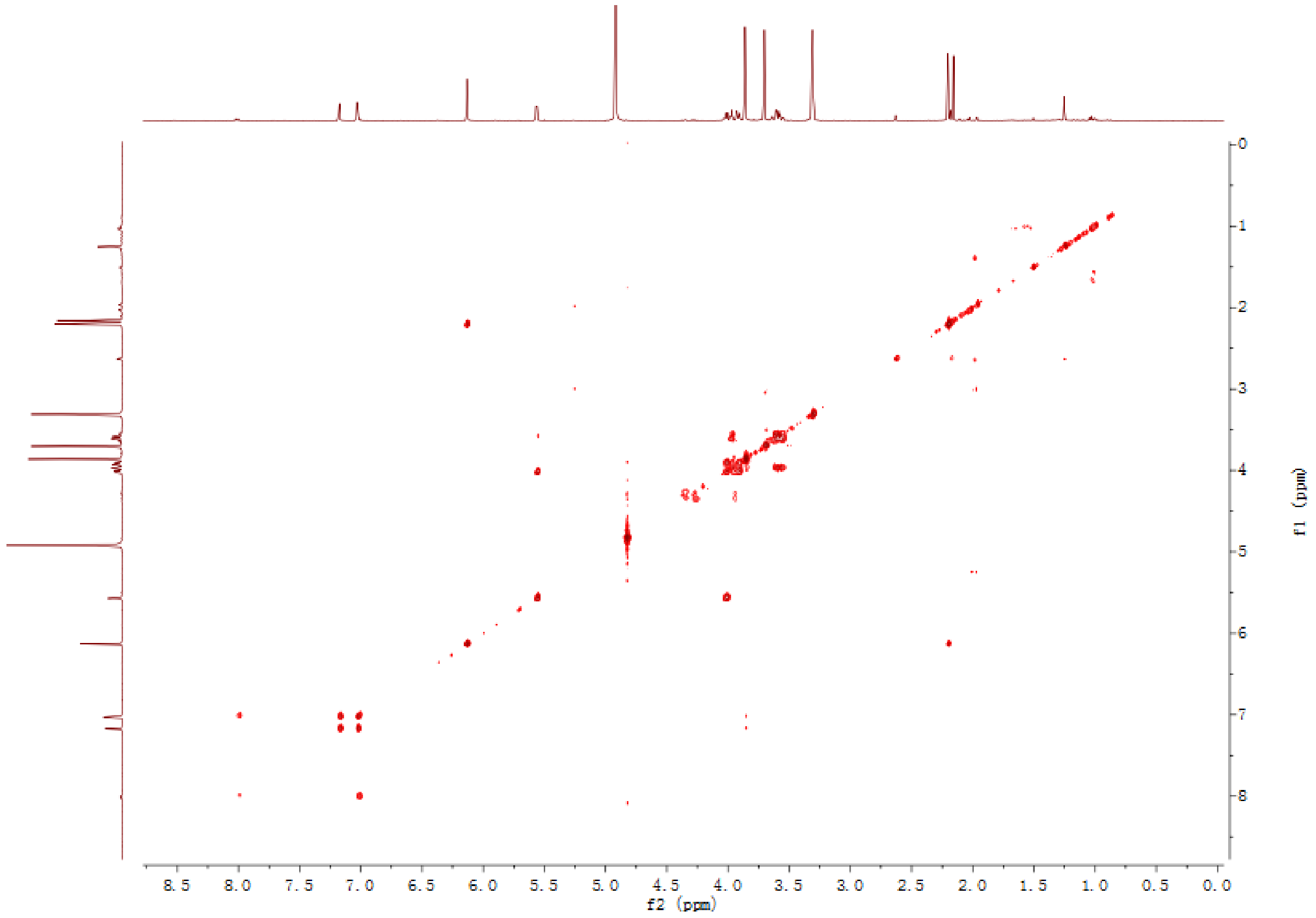

Fig. S12 HSQC spectrum of pestalotione C (**7**; 400 MHz, methanol-d_4)

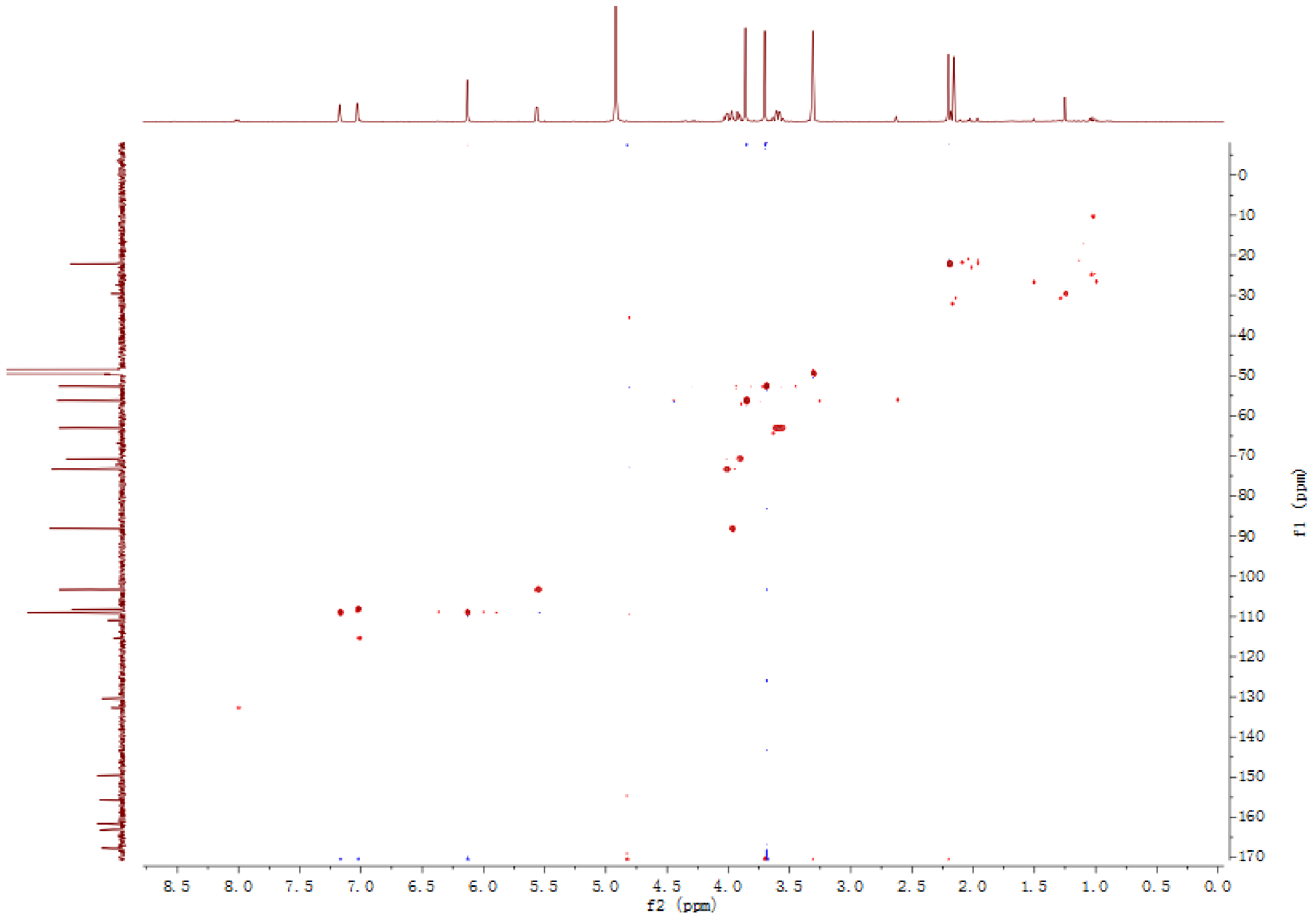

Fig. S13 HMBC spectrum of pestalotione C (**7**; 400 MHz, methanol-d_4)

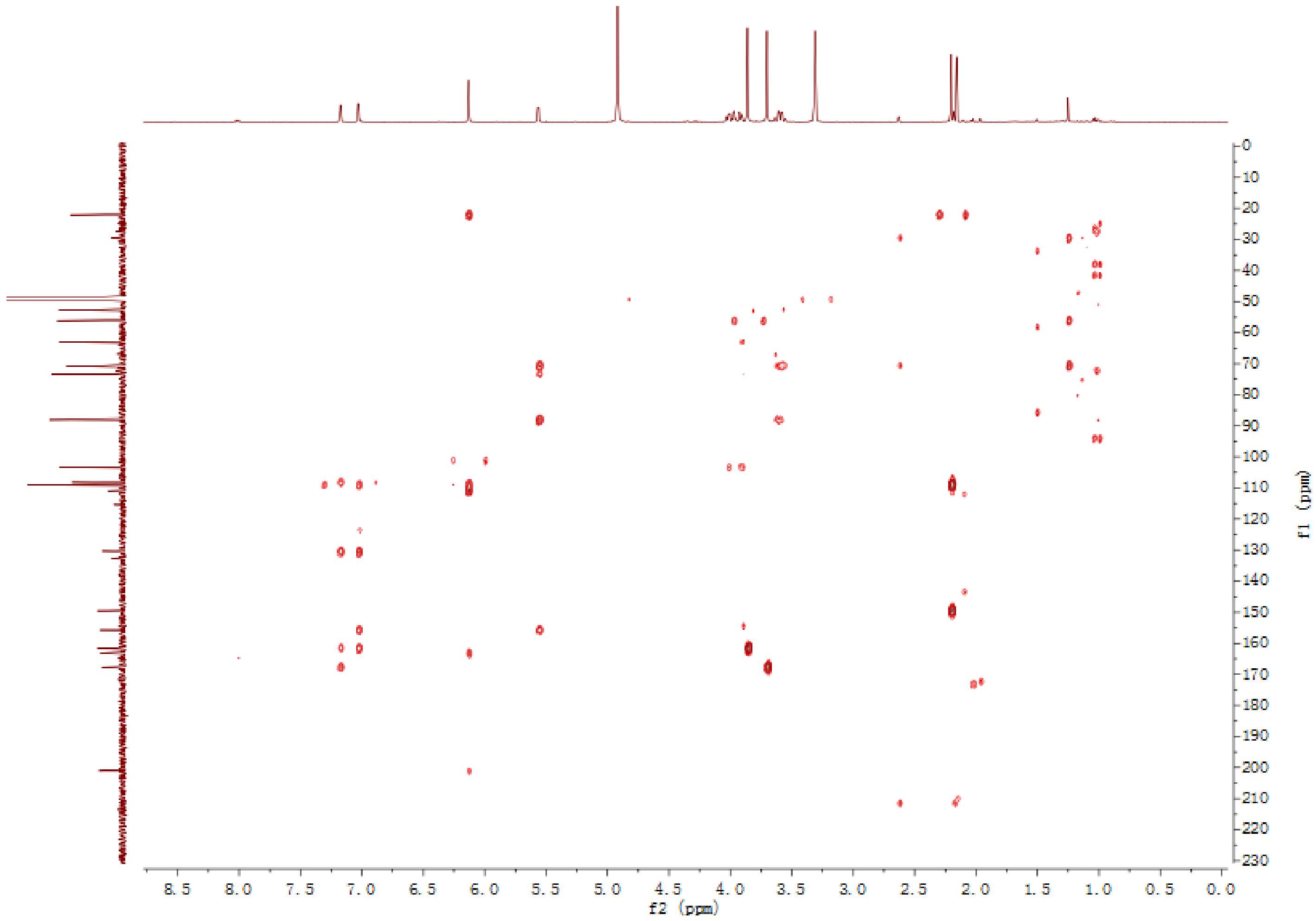

Fig. S14 ^{1}H NMR spectrum of pestalotione D (**8**; 400 MHz, $CDCl_3$)

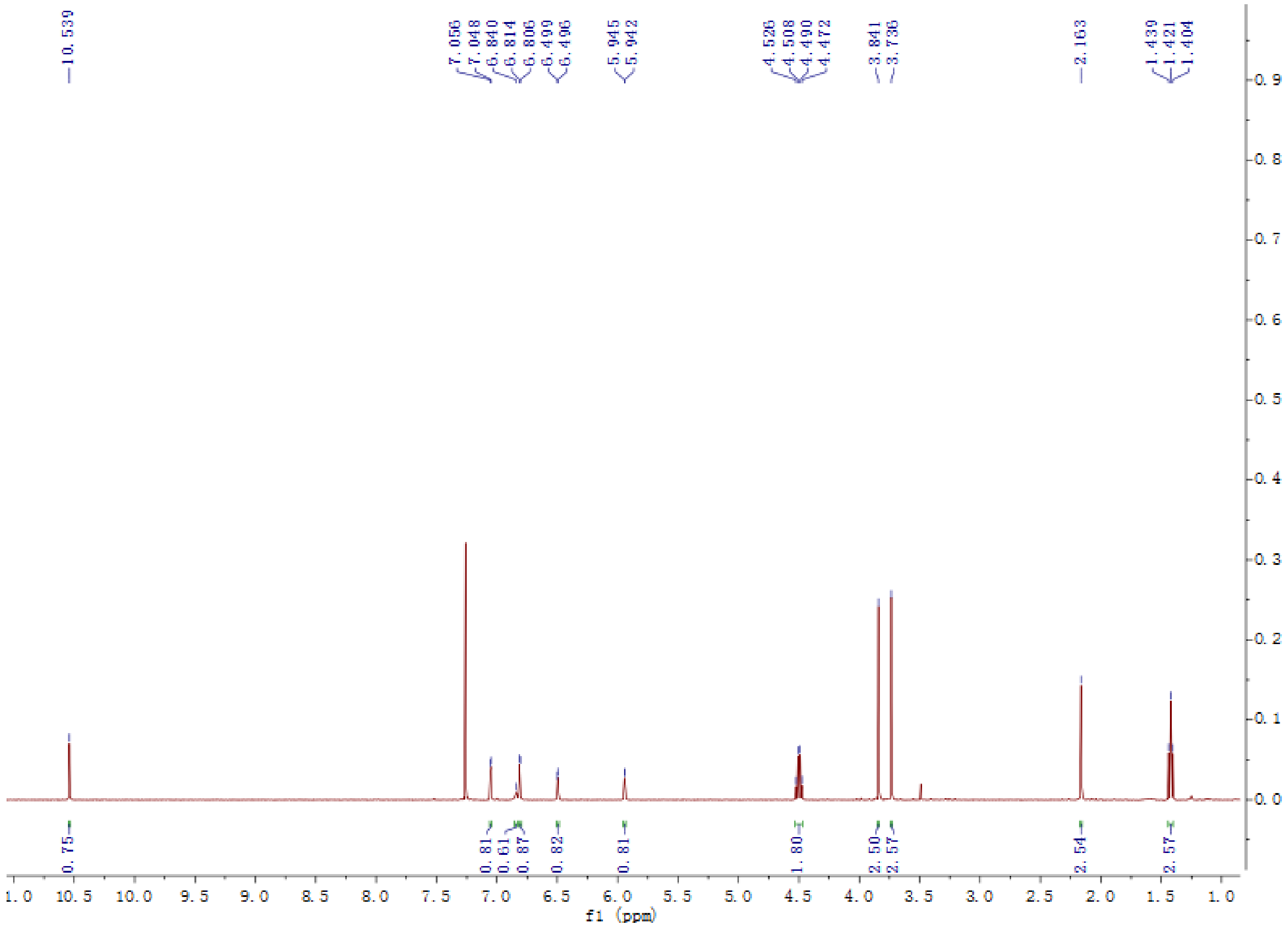

Fig. S15 ^{13}C NMR spectrum of pestalotione D (**8**; 100 MHz, $CDCl_3$)

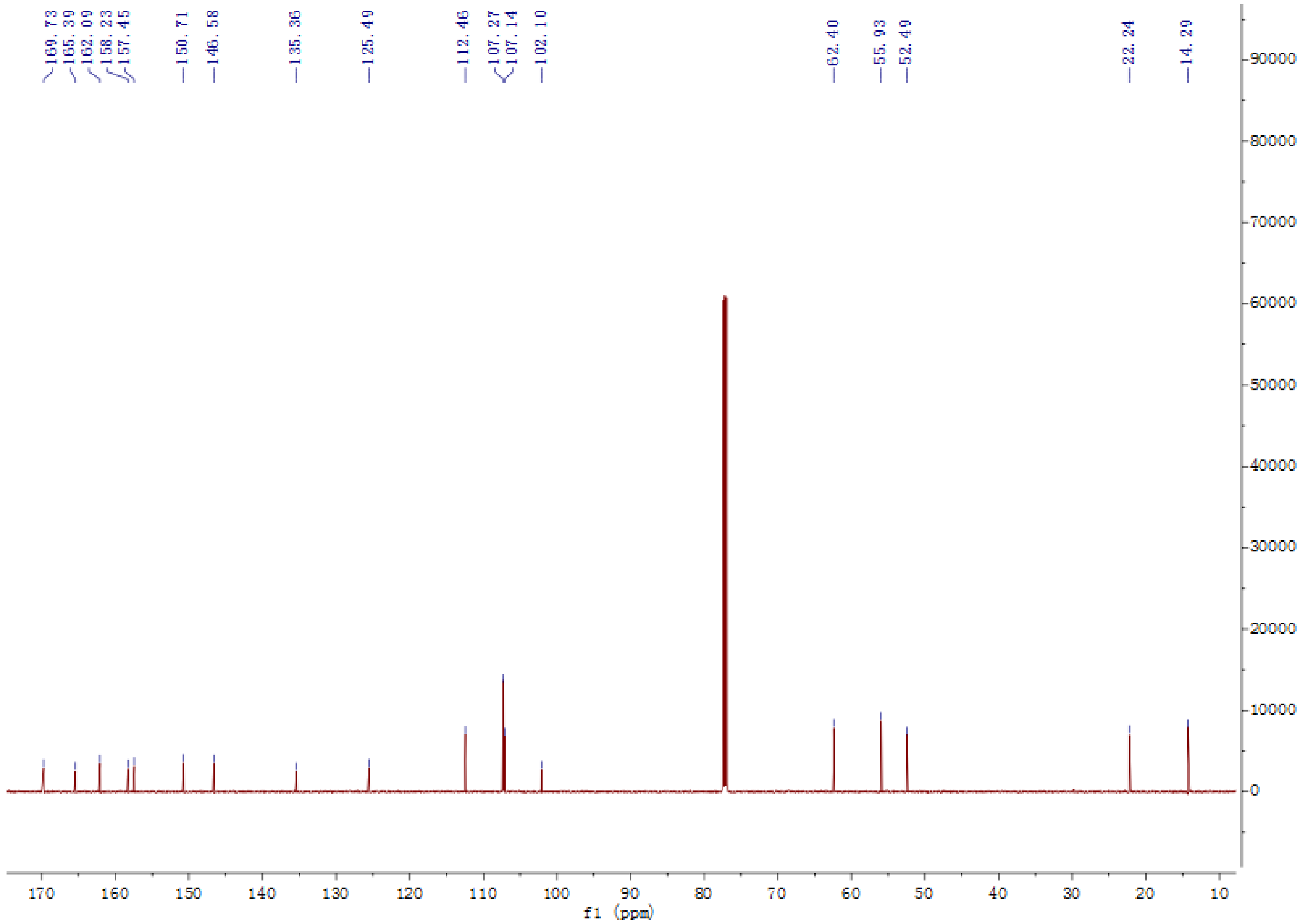

Fig. S16 HSQC spectrum of pestalotione D (**8**; 400 MHz, $CDCl_3$)

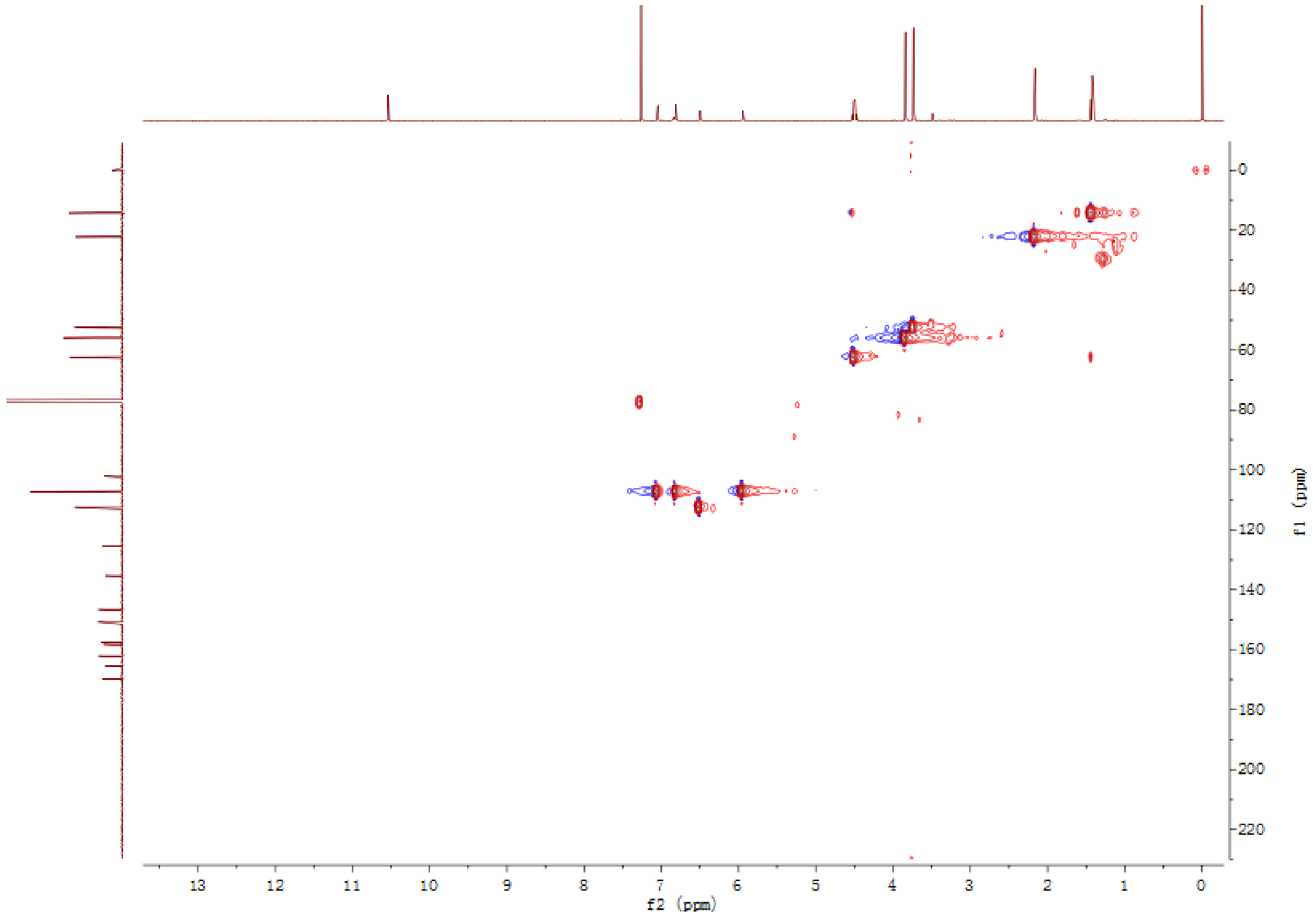

Fig. S17 HMBC spectrum of pestalotione D (**8**; 400 MHz, $CDCl_3$)

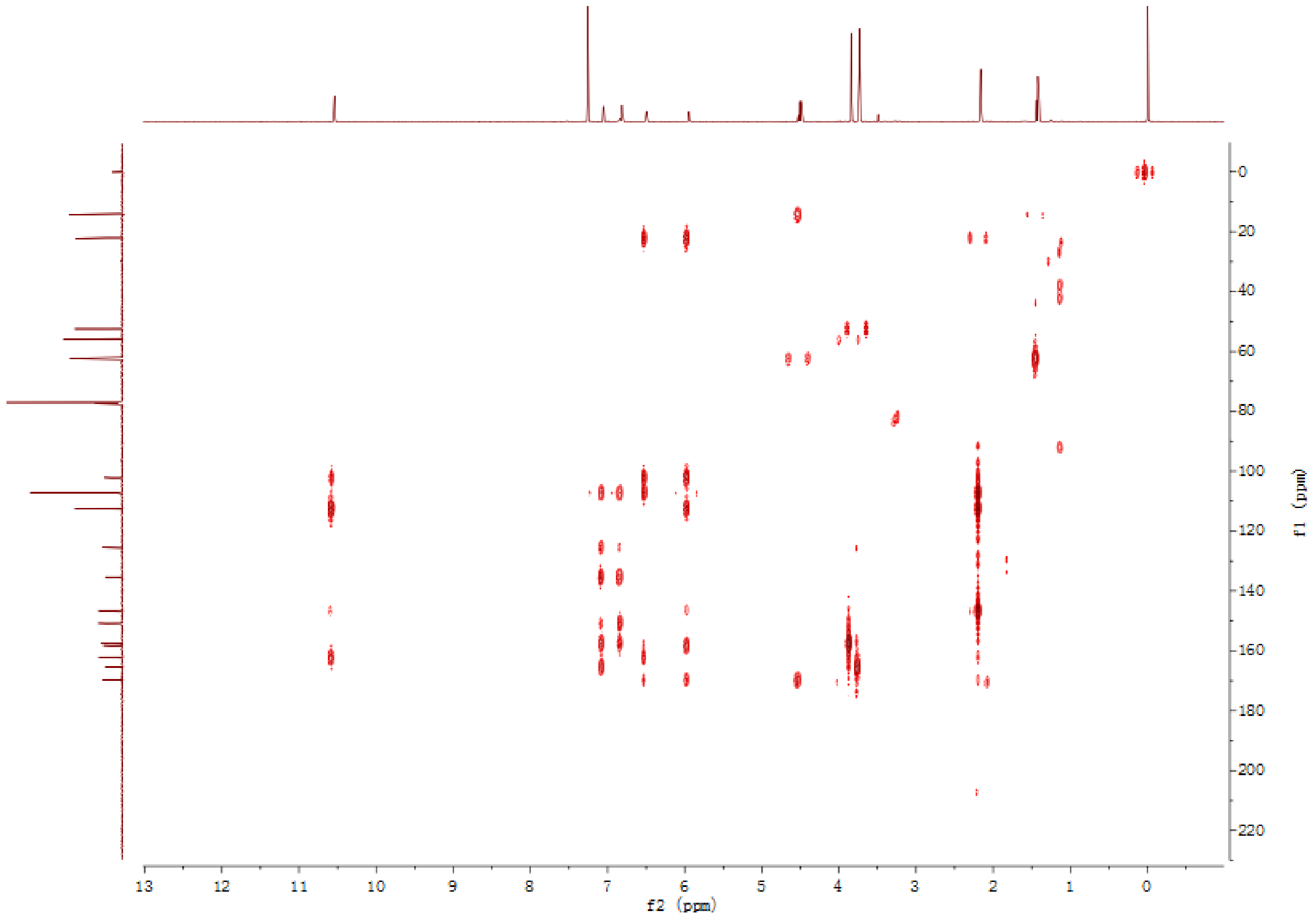

Supplement: Supplementary file 1 [file molecules-25-00470-s001.pdf]
